# Annexin A1 accounts for an anti-inflammatory binding target of sesamin metabolites

**DOI:** 10.1038/s41538-020-0064-6

**Published:** 2020-02-20

**Authors:** Yasuaki Kabe, Daisuke Takemoto, Ayaka Kanai, Miwa Hirai, Yoshiko Ono, Sota Akazawa, Manabu Horikawa, Yoshinori Kitagawa, Hiroshi Handa, Tomohiro Rogi, Hiroshi Shibata, Makoto Suematsu

**Affiliations:** 1 Department of Biochemistry, Keio University School of Medicine, Tokyo, 160-8582 Japan; 2 Japan Agency for Medical Research and Development, Core Research for Evolutional Science and Technology (AMED-CREST), Tokyo, Japan; 3 Institute for Health Care Science, Suntory Wellness Limited, 8-1-1 Seikadai, Seika, Soraku, Kyoto, 619-0284 Japan; 4 Bioorganic Research Institute, Suntory Foundation for Life Sciences (SUNBOR), 8-1-1 Seikadai, Seika, Soraku, Kyoto, 619-0284 Japan; 5 Department of Nanoparticle Translational Research, Tokyo Medical University, Tokyo, Japan

**Keywords:** Mechanism of action, Proteins

## Abstract

Sesamin [(7α,7′α,8α,8′α)-3,4:3′,4′-bis(methylenedioxy)-7,9′:7′,9-diepoxylignane] is a major lignan in sesame seeds. Sesamin is converted to the catechol metabolite, SC1 [(7α,7′α,8α,8′α)-3′,4′-methylenedioxy-7,9′:7′,9-diepoxylignane-3,4-diol] with anti-inflammatory effects after oral administration. However, its molecular target remains unknown. Analysis using high-performance affinity nanobeads led to the identification of annexin A1 (ANX A1) as an SC1-binding protein. SC1 was found to bind to the annexin repeat 3 region of ANX A1 with a high-affinity constant (Kd = 2.77 μmol L^−1^). In U937 cells, SC1 exhibited an anti-inflammatory effect dependent on ANX A1. Furthermore, administration of sesamin or SC1 attenuated carbon tetrachloride-induced liver damage in mice and concurrently suppressed inflammatory responses dependent on ANX A1. The mechanism involved SC1-induced ANX A1 phosphorylation at serine 27 that facilitates extracellular ANX A1 release. Consequently, the ANX A1 released into the extracellular space suppressed the production of tumor necrosis factor α. This study demonstrates that ANX A1 acts as a pivotal target of sesamin metabolites to attenuate inflammatory responses.

## Introduction

**Fig. 1:**
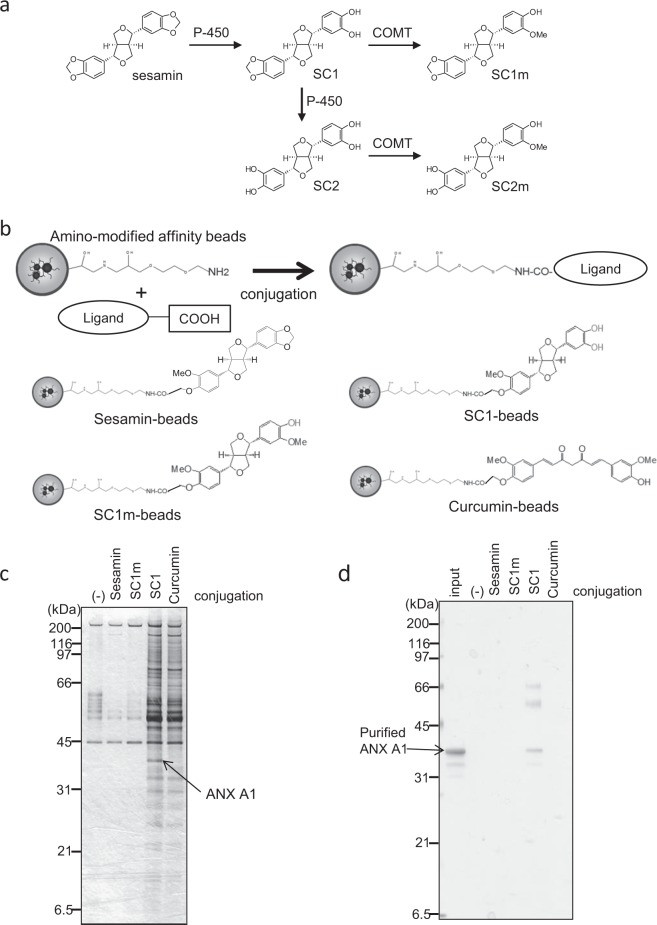
Identification of ANX A1 as a sesamin metabolite-binding protein using affinity nanobeads. **a** Scheme of sesamin metabolism in the liver. Sesamin is catalysed into SC1 and subsequently SC2 by cytochromes P450. SC1 or SC2 is further methylated into SC1m or SC2m by catechol *O*-methyl transferase (COMT). **b** Scheme of sesamin derivative-conjugation with affinity nanobeads. The carboxylated derivatives are conjugated with amino-modified nanobeads (upper panel). Lower panels show the chemical structures of the beads conjugating with sesamin, SC1, SC1m, or curcumin. **c** Affinity purification of the SC1-binding protein from U937 cell lysate. The affinity nanobeads conjugating with the ligands were incubated with U937 cell lysate, and bound proteins were analysed with SDS-PAGE before visualising using silver staining. The indicated protein (arrow) was identified as ANX A1 using peptide sequencing via ESI-MS. **d** Recombinant ANX A1 protein was incubated with affinity nanobeads, and bound proteins were analysed using SDS-PAGE before visualising with silver staining.

Sesame (*Sesamum indicum* L.), which is consumed as sesame seeds or oil, has been traditionally used as a natural remedy in Asia and the Middle East.^1^ Sesamin [(7α,7′α,8α,8′α)-3,4:3′,4′-bis(methylenedioxy)-7,9′:7′,9-diepoxylignane] is a major lignan in sesame seeds that is thought to potently attenuate inflammation, but the molecular mechanisms underlying its effects remain largely unknown. Sesamin administration has been shown to suppress tumor necrosis factor-α (TNFα) expression and liver injury in a carbon tetrachloride (CCl_4_)-induced acute^2^ and chronic^3^ hepatitis model in rodents. Furthermore, sesamin has been shown to suppress TNFα or monocyte chemotactic protein-1 (MCP-1) in the plasma of lipopolysaccharide (LPS)-treated mice^4^ and inhibit chemotaxis activation in human monocytes and mouse leucocytes.^5^ Sesamin intake improved liver function in a human study.^6^ Sesamin is metabolised in the liver.^7^ As shown in Fig. 1a, methylene dioxide groups of sesamin are catalysed by cytochromes P450 such as CYP2C9^8^ and converted to catechol-type metabolites, SC1 [(7α,7′α,8α,8′α)-3′,4′-methylenedioxy-7,9′:7′,9-diepoxylignane-3,4-diol] and SC2 [(7α,7′α,8α,8′α)-7,9′:7′,9-diepoxylignane-3,3′,4,4′-tetraol], respectively. The catechol group is further methylated to generate SC1m or SC2m via catechol *O*-methyl transferase.^7^ A pharmacokinetics study of sesamin in human blood showed that high concentrations of SC1, but not sesamin, were observed, indicating that sesamin is quickly converted into SC1 as a major metabolite.^9^ Recently, it was reported that SC1 exhibited a potent anti-inflammatory effect on LPS-induced nitric oxide (NO) production in monocytes compared to sesamin’s effect.^10^ However, the molecular mechanism of action of SC1 remains unclear.

Annexins are a multigene family of the Ca^2+^-regulated phospholipid-binding and membrane-binding proteins that bind Ca^2+^ at multiple EF hand motifs called annexin repeats.^11,12^ Annexin A1 (ANX A1) was first identified as an inducible factor, namely lipocortin 1, using glucocorticoids that could inhibit cPLA_2_.^13,14^ ANX A1 has a unique N-terminal region embedded within its core annexin repeat domain 3, which is exposed by binding with Ca^2+^.^14–16^ Furthermore, the N-terminal peptide of ANX A1 also exhibits anti-inflammatory effects.^17,18^ ANX A1 is known to be regulated by its translocation to the cell surface or extracellular secretion, which is induced by ANX A1 phosphorylation at the Ser27 residue via mitogen-activated protein kinase (MAPK), phosphoinositide 3-kinase, and protein kinase C (PKC)-dependent signalling pathways.^19^ Recently, several anti-inflammatory drugs like cromolyn were shown to exhibit anti-inflammatory activity by accelerating the extracellular release of ANX A1 in mast cells, leucocytes, or monocytes.^20–22^

We previously developed an application for high-performance affinity nanobeads, which enable the direct purification of binding proteins for small-molecule compounds.^23,24^ Using the beads, various receptor proteins have been identified for chemical compounds, including drugs, metabolites, or natural products.^25–28^ We recently identified the sesamin-binding protein in the sesame plant, and characterised its roles in plant growth.^29^ Here, we identified ANX A1 as a pivotal target protein for the sesamin metabolite, SC1. SC1 exhibits an anti-inflammatory effect by promoting phosphorylation and extracellular ANX A1 release. Furthermore, ANX A1 is required for sesamin’s hepato-protective effect in a mouse model of CCl_4_-induced liver injury.

## Results

### Identification of ANX A1 as the SC1-binding protein

Affinity nanobeads were used here to identify the binding proteins of sesamin derivatives. For conjugating with the amino-modified affinity nanobeads, we prepared the derivatives introducing the carboxyl group for sesamin, SC1, SC1m, or curcumin used as a negative control polyphenol compound, respectively (Fig. S1). As shown in the schematic diagram in Fig. 1b, these compounds were conjugated using amine coupling to the beads. Using these beads, we attempted to purify the proteins binding to sesamin derivatives from human leukaemia U937 cell extract (Fig. 1c). Among several proteins observed, a protein with a molecular weight of ~37 kDa specifically bound to the SC1 beads. This band was identified as ANX A1 using electrospray ionisation-mass spectrometry (ESI-MS) analysis. To confirm this, we prepared the purified recombinant ANX A1 protein expressed in *Escherichia coli*. As shown in Fig. 1d, recombinant ANX A1 was bound to SC1 beads. In addition, no binding was observed when using recombinant ANX V (Fig. S2a), suggesting that ANX A1 is a specific binding protein for SC1.

**Fig. 2:**
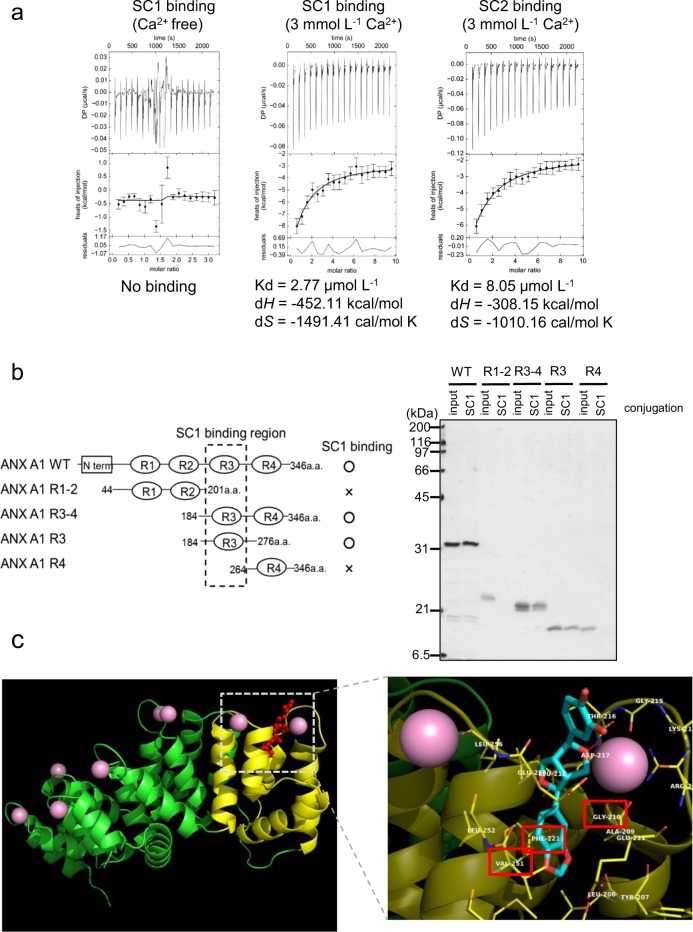
Analyses of binding property between ANX A1 and sesamin metabolites. **a** Analyses of binding affinity between ANX A1 and sesamin metabolites using isothermal titration calorimetry (ITC). The binding affinity between ANX A1 and SC1 with ITC was analysed in the presence of EDTA (Ca^2+^ free, left panel) or CaCl_2_ (middle panel). Right panel showed the binding affinity between ANX A1 and SC2 using CaCl_2_-containing buffer. The binding values (Kd, Δ*H*, or Δ*S*) were calculated using the SEDPHAT program, and the data were shown as the average of three independent experiments. **b** Identification of the SC1-binding domain of ANX A1. The SC1-conjugating beads were incubated with wild-type (WT) or deletion mutant proteins of ANX A1 (left panel). Bound proteins were analysed with SDS-PAGE before visualising using silver staining (right panel). **c** Docking simulation of ANX A1 and SC1. ANX A1 is depicted as green or yellow (repeat 3 domain) ribbon (left panel). Ca^2+^ is depicted as pink balls. SC1 is shown as red sticks and balls. Close-up view of binding pocket of SC1 (light blue) at the Ca^2+^-binding region of ANX A1 repeat 3 domain (right panel). The amino acid residues marked by red squares (Gly210, Phe221 or Val251) indicate sites used for binding analysis of ANX A1 point mutants (Supplementary Fig. 3).

The structural conformation of ANX A1 is changed by binding with Ca^2+^ on its annexin repeat regions. We examined whether SC1 binding to ANX A1 was Ca^2+^-dependent. While apo-ANX A1 treated with ion chelator EDTA was hardly able to bind SC1 beads, the Ca^2+^ ANX A1 form could bind SC1 beads (Fig. S2b). Binding analysis with isothermal titration calorimetry (ITC) revealed that SC1 bound to the Ca^2+^ form of ANX A1, but not apo-ANX A1, and the binding affinity (Kd) was calculated as 2.77 μmol L^−1^ (Fig. 2a). Furthermore, sesamin derivative SC2 also bound to the Ca^2+^ form of ANX A1 with a binding affinity (Kd) of 8.05 μmol L^−1^. We were unable to detect any binding activity between sesamin and ANX A1 using ITC analysis. ANX A1 contains four Ca^2+^-binding motifs (annexin repeats). A binding assay was also performed to determine the binding domain of SC1 using deletion mutant proteins of ANX A1 (Fig. 2b). The SC1 beads bound to the ANX A1 proteins containing the repeat 3 (R3) domain (WT, R3–4, or R3), but the binding activity disappeared when using mutants lacking the R3 domain (R1–2, or R4), suggesting that SC1 specifically recognised R3 domain of ANX A1.

We next performed three‐dimensional computational modelling analysis to identify the possible interaction modes between SC1 and ANX A1 (Fig. 2c). The analysis revealed that SC1 bound to a pocket on the R3 domain of ANX A1, and the catechol group of SC1 was associated with Ca^2+^ of ANX A1 R3 via hydrogen bonding. To confirm this binding model, ITC binding analysis was performed using recombinant ANX A1 mutated in Gly210 (G210W), Phe221 (F221S), or Val251 (V251W), which is located in the SC1-binding pocket on R3. As shown in Fig. S3, all ANX A1 mutants (G210W, F221S, and V251W) disrupted the binding activity to SC1 in contrast with ANX A1 WT binding, suggesting that SC1 binds to the pocket on the R3 domain of ANX A1.

### SC1 exhibits an anti-inflammatory effect dependent on ANX A1

**Fig. 3:**
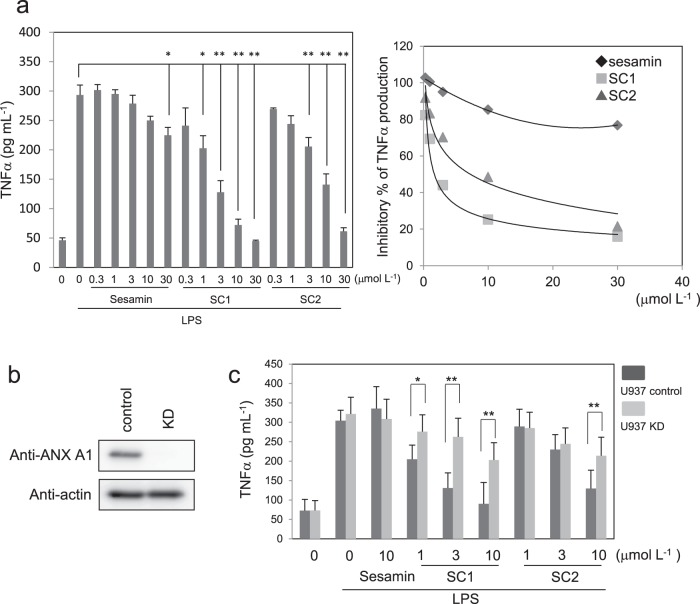
SC1 suppresses TNFα production dependent on ANX A1 in U937 cells. **a** Suppressive effect of sesamin derivatives on TNFα production in U937 cells. U937 cells were differentiated by adding PMA for 24 h and treating with or without 1 µg mL^−1^ LPS and/or the indicated amount of sesamin, SC1 or SC2 for 12 h. TNFα production was analysed using ELISA. Right panel shows the relative inhibitory percentage of TNFα production by sesamin derivatives. **b** Protein expression of ANX A1 knockdown (KD) U937 cells. Control and shRNA for ANX A1 introducing lentivirus were infected into U937 cells. The cell lysates were analysed using western blotting using antibody against ANX A1 or β-actin. **c** U937 cells (control or ANX A1 KD) were differentiated with PMA and treated with or without 1 µg mL^−1^ LPS and/or the indicated amount of sesamin, SC1 or SC2. TNFα production was analysed using ELISA. All data represent the mean ± SD (*n* = 3). *p < 0.05, **p < 0.01 using unpaired Student’s *t* test.

ANX A1 has been reported to play a role in several anti-inflammatory activities.^15,30^ We first examined the effect of sesamin derivatives on TNFα production in U937 cells. TNFα production induced by LPS and phorbol 12-myristate 13-acetate (PMA) was significantly inhibited by adding SC1 and SC2 in a dose-dependent manner, while little inhibitory effect was observed with sesamin (Fig. 3a). The inhibitory rate (IC_50_) was calculated as IC_50_ of SC1 = 2.2 μmol L^−1^ and IC_50_ of SC2 = 10.3 μmol L^−1^, respectively (Fig. 3a, right panel). These concentrations were comparable to the binding constant with ANX A1 determined using ITC analysis. To examine whether ANX A1 contributes to the anti-inflammatory effect of sesamin derivatives, we generated stable U937 cells by knocking down ANX A1 expression (KD) using a lentivirus that introduced a short hairpin RNA (shRNA) that targets the ANX A1 sequence. Figure 3b showed that ANX A1 expression was significantly reduced by ANX A1-KD compared to control U937 cells, which were infected with a lentivirus lacking the targeting sequence. The TNFα production in the control U937 cells was inhibited by adding either SC1 or SC2. However, the inhibitory effects of SC1 or SC2 disappeared in the U937 cells with knocked down ANX A1 (Fig. 3c), suggesting that SC1 and SC2 inhibit the TNFα production via ANX A1-mediated mechanisms.

### SC1 elicits an anti-inflammatory effect by promoting the phosphorylation and extracellular release of ANX A1

**Fig. 4:**
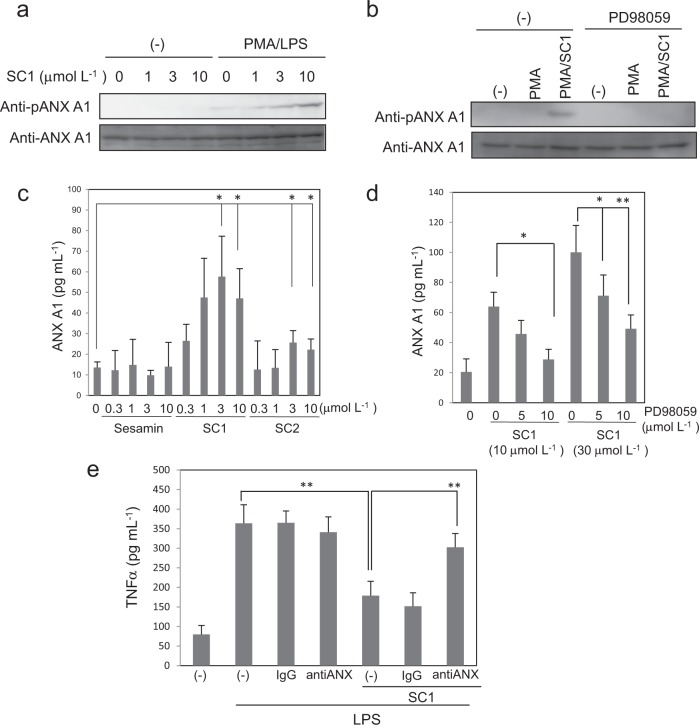
SC1 elicits an anti-inflammatory effect by promoting the phosphorylation and extracellular release of ANX A1. **a**, **b** U937 cells treated with or without PMA and LPS were incubated with the indicated amount of SC1 for 12 h (**a**), and the cell lysates were analysed using western blotting with antibody against ANX A1 or phosphorylated (Ser27) ANX A1. MAPK inhibitor PD98059 was treated 1 h prior to LPS treatment (**b**). **c**, **d** Extracellular release of ANX A1 from U937 cells was detected using the ELISA assay as mentioned above. PD98059 was treated 1 h prior to LPS treatment (**d**). **e** U937 cells were treated with control IgG or anti-ANX A1 antibody 1 h before LPS treatment in the presence or absence of 10 μmol L^−1^ SC1. The TNFα production in cell culture media was analysed using ELISA. All data represent the mean ± SD (*n* = 3). *p < 0.05, **p < 0.01 using unpaired Student’s *t* test.

It has been reported that ANX A1 serine residue phosphorylation at the N-terminal region enhanced its extracellular release followed by an up-regulation of the anti-inflammatory activity.^20,21^ We examined the effect of ANX A1 phosphorylation by SC1 using an antibody against the phosphorylated ANX A1 at the Ser27 residue. As shown in Fig. 4a, while ANX A1 phosphorylation was not observed without PMA and LPS stimulation, SC1 significantly induced phosphorylation after PMA and LPS treatment in a dose-dependent manner. The ANX A1 phosphorylation is known to be induced via a PKC-dependent pathway.^20^ The MEK inhibitor PD98059, which is a downstream kinase of the PKC pathway, suppressed the SC1-induced phosphorylation of ANX A1 (Fig. 4b). Next, we examined the effect of extracellular ANX A1 release, by sesamin derivatives. Figure 4c showed that the cellular release of ANX A1 was significantly enhanced by treatment with SC1 or SC2, but not with sesamin. The up-regulation of ANX A1 release was inhibited by PD98059 in a dose-dependent manner (Fig. 4d). The released ANX A1 has an anti-inflammatory effect. We next examined whether ANX A1 released into the extracellular space contributed to the anti-inflammatory effect of SC1. TNFα production by SC1 was suppressed by adding the antibody that recognised the ANX A1 N-terminal region. These results suggested that SC1 suppressed TNFα production by stimulating ANX A1 phosphorylation and extracellular release.

### ANX A1 is required for the hepato-protective effects of sesamin and SC1

**Fig. 5:**
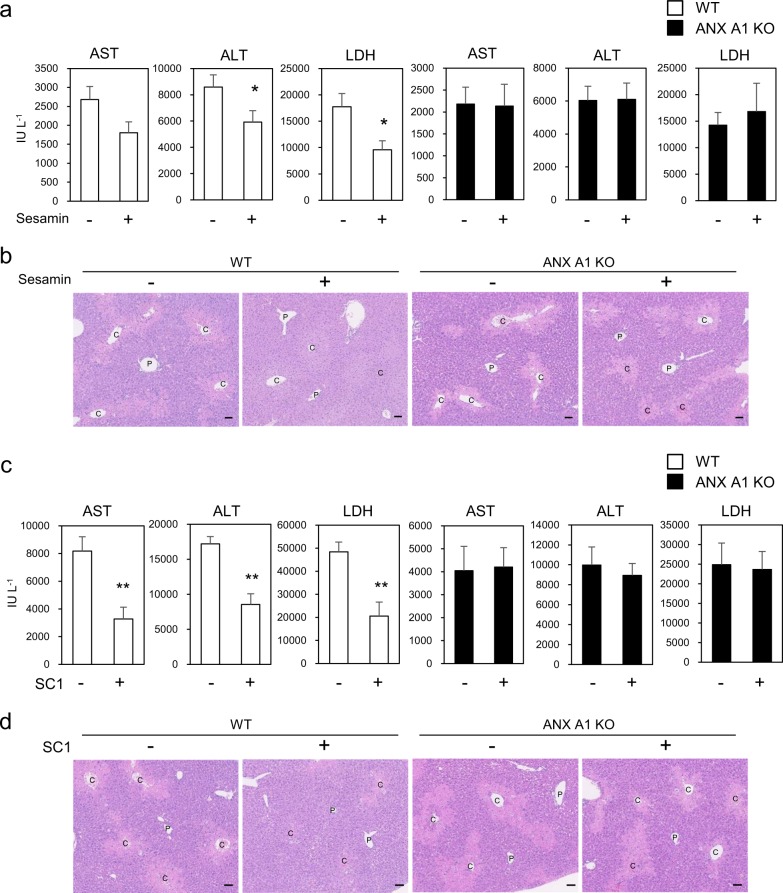
ANX A1 is required for the hepato-protective effects of sesamin and SC1 in the CCl_4_-induced liver damage model. **a**, **b** Effect of sesamin administration on the CCl_4_-induced liver damage in WT or ANX A1-KO mice. WT or ANX A1-KO mice (C57BL/6J) were intraperitoneally administered CCl_4_, and sesamin (25 mg kg^−1^ body weight), or vehicle was orally administered twice at 1 h before and 7 h after CCl_4_ administration. Hepatic damage marker enzymes (AST, ALT or LDH) in the plasma were measured 24 h after CCl_4_ administration (**a**). Data represent the mean ± SE. *n* *=* 13–15 mice in each group. *p < 0.05 using unpaired Student’s *t* test. The liver section was histologically evaluated using H&E staining (**b**). P and C indicated the portal and central vein, respectively. Scale bar: 50 μm. **c**, **d** Effect of SC1 on the CCl_4_-induced liver damage in WT or ANX A1-KO mice. SC1 (50 mg kg^−1^ body weight) or vehicle was intraperitoneally administered twice at 1 h before and 7 h after CCl_4_ administration. AST, ALT and LDH in the plasma, 24 h after CCl_4_ administration, were measured (**c**). Data represent the mean ± SE. *n* *=* 9–14 mice in each group. **p < 0.01 using unpaired Student’s *t* test. (**d**) The liver section was histologically evaluated using H&E staining. Scale bar: 50 μm.

We generated ANX A1-knockout (KO) mouse using homologous recombination to further examine the anti-inflammatory effects of sesamin and SC1 in vivo (Fig. S4a). Deletion of ANX A1 expression in the liver was confirmed using western blotting and quantitative PCR (qPCR) (Fig. S4b, c). Oral administration of sesamin (one administration at pre-injection and one administration post-injection of CCl_4_) significantly suppressed the elevated hepatic enzymes alanine aminotransferase (ALT) or lactate dehydrogenase (LDH) in CCl_4_-treated mouse plasma (Fig. 5a, left panels). In contrast, these suppressive effects were not observed in ANX A1-null mice (Fig. 5a, right panels). Histological analyses of the liver tissues confirmed that sesamin suppressed liver injury around the central vein in WT mice, but not in ANX A1-null mice (Fig. 5b).

Orally administered sesamin is metabolised into SC1 as a major metabolite by cytochrome P450.^9^ We used liquid chromatography–mass spectrometry analysis to confirm that 8 to 12 μmol L^−1^ of SC1 was detected in livers of mice that received an oral administration of sesamin (Fig. S5). We showed that sesamin metabolite SC1, but not sesamin, exhibits its anti-inflammatory activities in cells. To examine the effect of SC1 in vivo, we analysed the effect on the CCl_4_-induced liver injury by administering SC1 to mice. SC1 suppressed aspartate aminotransferase (AST), ALT and LDH levels in the plasma of CCl_4_-treated WT mice, but not in the ANX A1-null mice (Fig. 5c). Histological analyses were also consistent with these effects (Fig. 5d). Thus, these results indicated that ANX A1 is required to suppress CCl_4_-induced liver injury using sesamin or SC1.

### Sesamin suppresses hepatic inflammatory responses dependent on ANX A1

**Fig. 6:**
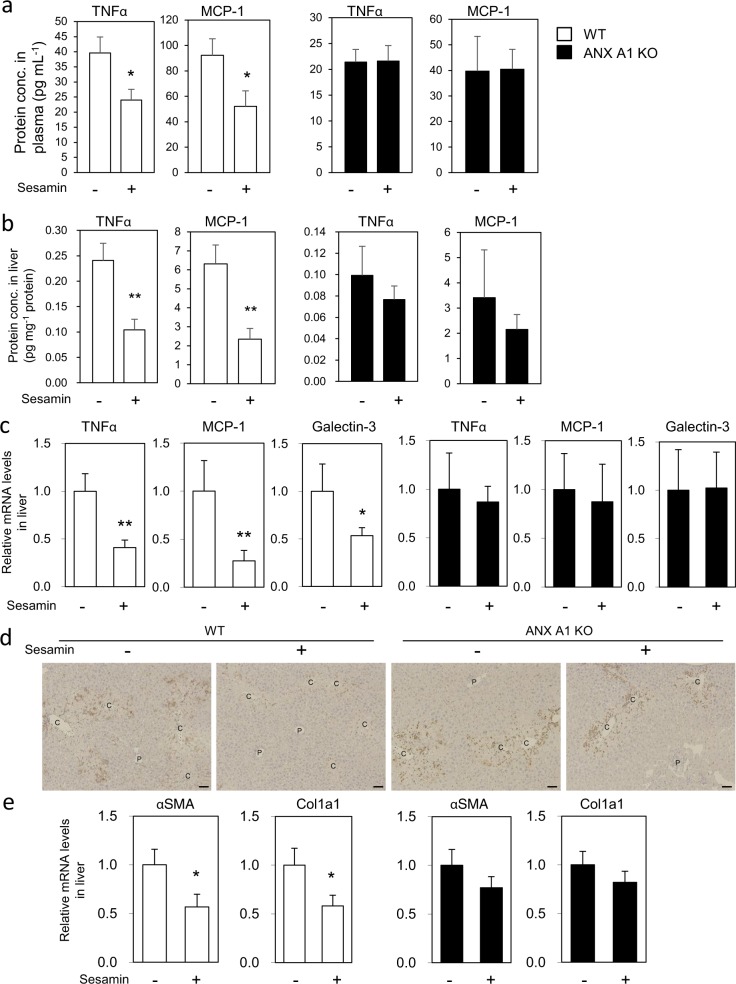
ANX A1 is required for the suppressive effects of sesamin against CCl_4_-induced liver inflammation. Effect of sesamin on CCl_4_–induced inflammation induced in WT or ANX A1-KO mice. WT or ANX A1-KO mice were intraperitoneally injected with CCl_4_. Sesamin (25 mg kg^−1^ body weight) or vehicle was administered twice at 1 h before and 7 h after CCl_4_ administration. The plasma or liver were collected for analysis 12 h after CCl_4_ administration. Production of TNFα and MCP-1 protein in the plasma (**a**) or in the liver (**b**) was analysed using ELISA. **c** Expression of TNFα, MCP-1, and galectin-3 mRNA in the liver was measured using qPCR, and normalised to 18S ribosomal RNA expression. The graph showed as the relative fold change compared to mRNA expression in the liver treated with vehicle. Data represent the mean ± SE. *n* = 5-10 mice in each group. *p < 0.05 or **p < 0.01 using unpaired Student’s *t* test. **d** The liver section collected 24 h after CCl_4_ administration with or without sesamin treatment was stained using galectin-3 antibody. Scale bar: 50 μm. **e** mRNA expression of αSMA or Col1a1 was measured by qPCR from the liver tissues collected 24 h after CCl_4_ administration with or without treatment of sesamin, and normalised by the expression of 18S rRNA. Data represent the mean ± SE. *n* = 13–15 mice in each group. *p < 0.05 using unpaired Student’s *t* test.

Next, we examined whether ANX A1 is required for the anti-inflammatory effects of sesamin in CCl_4_-administered mice. Administration of sesamin significantly suppressed the levels of TNFα and MCP-1 proteins in the plasma (Fig. 6a) and livers (Fig. 6b) of WT mice, but not in those of the ANX A1-null mice. We found that SC1 suppressed TNFα and MCP-1 expression in the livers of WT mice treated with CCl_4_, but no effect was observed in ANX A1-null mice (Fig. S6). We also showed that sesamin administration significantly suppressed messenger RNA (mRNA) expression of TNFα, MCP-1, and galectin-3, which is a marker for activated monocytes, in the WT mice liver, but these effects were not observed in ANX A1-null mice (Fig. 6c). Furthermore, histological analysis of the liver tissues revealed that sesamin suppressed the accumulation of galectin-3-positive cells, which indicates infiltration of activated monocytes residing in and around the central veins in the WT mice, but not in the ANX A1-null mice (Fig. 6d). In addition, administration of sesamin suppressed liver mRNA expression of α-smooth muscle actin (αSMA) and collagen α-1(I) (Col1a1), which are liver fibrosis markers, in the CCl_4_-treated WT mice, but not in the ANX A1-null mice (Fig. 6e). These results suggested that ANX A1 is required for the anti-inflammatory effect of orally administered sesamin in mice with CCl_4_-induced liver injury.

## Discussion

This study showed that the sesamin metabolite SC1 or SC2 suppressed TNFα production in U937 cells via mechanisms involving ANX A1, while no inhibitory effect was observed using sesamin by itself. Being in good agreement with these results, SC1 or SC2 enabled ANX A1 binding, but any binding activity was seen between sesamin and ANX A1. Previous reports have shown that sesamin exhibits anti-inflammatory effects like metalloproteinase-9,^31^ interleukin-6,^32^ or cyclooxygenase-2 suppression,^33^ but a high dose of sesamin (around 50 to 100 μmol L^−1^) is necessary for these effects. It has recently been reported that SC1 strongly inhibited the expression of inducible NO synthase (iNOS) compared to sesamin.^10^ Interestingly, this study also showed that monocytes secrete β-glucuronidase to generate SC1 from SC1-glucuronides, which are SC1-metabolites generated in liver,^7^ leading to the suppression of the iNOS expression.^10^ This suggests that SC1 act as a major active metabolite for anti-inflammatory activity in monocytes, upon sesamin administration. In the in vivo study, administration of sesamin or SC1 exhibited hepato-protective and anti-inflammatory activity in the CCl_4_-induced liver injury mouse model (Figs. 5 and 6). Oral administration of sesamin in mice resulted in SC1 production at a concentration of around 10 μmol L^−1^ in the liver (Fig. S5). Our results indicated that sesamin, which has no anti-inflammatory effect on its own, functions as a pro-active form that exhibits anti-inflammatory and hepato-protective effects by metabolising in the liver to produce the active metabolite SC1.

SC1 directly binds to ANX A1 and promotes the phosphorylation of ANX A1 at the N-terminal Ser27 residue, facilitating its extracellular release. ANX A1 consists of four Ca^2+^ binding repeat domains at the central core region and an active N-terminal region.^34,35^ The structural analyses of ANX A1 revealed that the N-terminal region is embedded by the annexin R3 domain. After binding the calcium ion, the conformation of the R3 is converted to eject the N-terminal domain.^36–38^ It has been suggested that the conformation change of the N-terminal domain in response to calcium ion accelerates the phosphorylation at ANX A1 Ser27 mediated by the PKC and MAPK pathways.^19,39^ The phosphorylated ANX A1 induces its cellular translocation and extracellular release.^19^ Our docking study revealed that SC1 binds to the Ca^2+^-bound form of ANX A1 at the annexin R3 domain. Although the complete mechanism for roles of the structural regulation by SC1 in the functional outcome of ANX A1 remains unknown, it is not unreasonable to suggest that the SC1 binding with the R3 domain of ANX A1 contributes to its conformation change to expose the N-terminal domain, resulting in phosphorylation activation at Ser27. It has been reported that the N-terminal region of ANX A1 or the N-terminal peptides exhibit anti-inflammatory action in leucocytes or monocytes by regulating a family of G protein-coupled receptors as formyl peptide receptors (FPRs), including FPR1, FPR2 and FPR3.^14,16^ While further studies are necessary to reveal anti-inflammatory mechanisms of ANX A1, the currents studies using sesamin metabolites shed light on new anti-inflammatory roles of ANX A1 released to the extracellular space that suppresses TNFα production via signalling pathways involving FPRs. In addition, our analyses showed that any effect for TNFα expression was not observed in the ANX A1 knockdown (KD) cells or KO mice. Although the exact reason has been unclear, several previous studies indicated that suppression of endogenous ANX A1 expression by KD or KO exhibits only a weak effect on TNFα expression.^40–42^ Supporting our results, Tang et al.^43^ showed that anaesthetic propofol suppressed TNFα expression by upregulating ANX A1, whereas no effect was observed using ANX A1 KD. These suggest that further activation of ANX A1 by inducers like sesamin metabolites might be required to exhibit a potent anti-inflammatory effect by ANX A1. Furthermore, in the CCl_4_-induced liver damage model, the level of hepatic injury markers or inflammatory cytokines was reduced in ANX A1-KO mice, compared with WT mice. Although the exact reason has been unclear, recent reports suggested that suppression of ANX A1 expression interferes with apoptotic cell death in several cell lines.^44–46^ These suggest that systematic disruption of ANX A1 expression in mice may contribute to protect against the CCl4-induced cell death in the liver via distinct mechanisms from ANX A1 activation by sesamin metabolites.

**Fig. 7:**
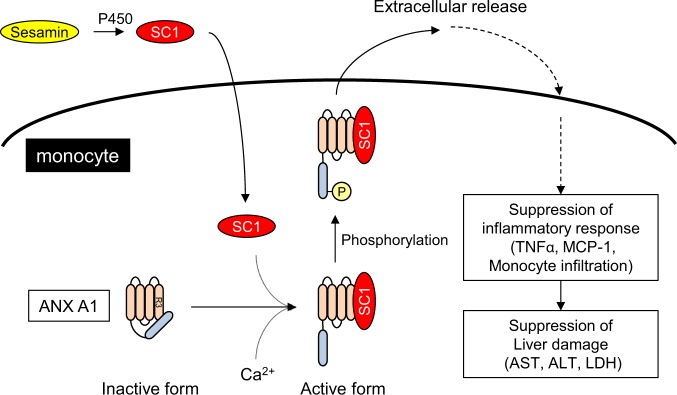
Schematic representation of the anti-inflammatory effect of SC1 mediated by ANX A1 activation. SC1 is produced by metabolising sesamin in hepatocytes. In monocytes, SC1 directly binds to the repeat 3 domain of Ca^2+^ form ANX A1. Under stimulated conditions like PMA/LPS in monocytes, SC1 promotes the phosphorylation of ANX A1 Ser27 and the subsequent extracellular release of ANX A1, and thereby eliciting an anti-inflammatory effect via the suppression of TNFα or MCP-1 production.

Based on the current results, a model for the mechanism of action of SC1 in monocytes is illustrated (Fig. 7). SC1 is generated by the metabolism of administered sesamin in hepatocytes. In monocytes, SC1 activates ANX A1 by facilitating the phosphorylation and subsequent extracellular secretion via direct binding with the R3 domain of Ca^2+^-bound ANX A1. SC1 exhibits anti-inflammatory activity by suppressing TNFα production by released ANX A1. The ANX A1-mediated anti-inflammatory activity by SC1 improves CCl_4_-induced liver injury by suppressing cytokine production and monocyte infiltration. In addition, we also found that sesamin suppressed the expression of liver fibrosis markers like αSMA and Col1a1, suggesting that sesamin intake might improve chronic hepatitis dependent on ANX A1. Recent studies suggested that ANX A1 is a potential drug target with anti-inflammatory effects.^17,18^ Treatment with ANX A1 N-terminal peptide fragment Ac2-26 protects against several inflammatory activities or disease models like ischaemia–reperfusion-induced acute lung injury,^47^ mast cell activation,^22^ atherosclerosis,^48^ gastric mucosal injury,^49^ or myocardial infarction.^50^ Further analysis may provide new insights into the beneficial effects of sesamin or SC1 administration, mediated by ANX A1, against various diseases.

## Methods

### Materials

Sesamin was isolated from sesame seeds.^51^ LPS and MAPK inhibitor PD98059 were purchased from Calbiochem (San Diego, CA, USA). CCl_4_ was purchased from Wako chemical (Tokyo, Japan). Purified olive oil and corn oil were purchased from Nacalai Tesque (Kyoto, Japan). Specific antibodies against ANX A1 or β-actin were purchased from Abcam or Sigma, respectively. Polyclonal antibodies against phosphorylated ANX A1 were generated using immunisation in rabbits with the ANX A1 peptide phosphorylated at the Ser27 residue (COSMO Bio., Tokyo, Japan).

### Preparation of sesamin metabolites

Sesamin metabolites SC1 and SC2 were prepared using chemical synthesis.^52^ Metabolites were isolated, purified as a single compound and the chemical structures were confirmed using nuclear magnetic resonance. The following compounds were used in this study: sesamin, SC1 and SC2.

### Plasmid constructions

Human ANX A1 complementary DNA (cDNA) was cloned from the U937 cell cDNA library (ATCC CRL-1593.2). The ANX A1 cDNA fragment, full-length (1–346 a.a.) or deletion mutants (R1–2: 44–201 a.a., R3–4: 184–346 a.a., R3: 184–276 a.a., or R4: 264–346 a.a.) were amplified using PCR, digested with BamHI and SalI, and ligated into pGEX-6P-1 (GE Healthcare, Massachusetts, USA) using primers shown in the Supplementary Methods.

### Preparation of recombinant proteins

BL21 (DE3) cells (Takara, Japan) were transformed with the pGEX-ANX A1 expression vectors for full-length or mutants, and the bacteria were incubated in LB with ampicillin at 37 °C until the optical density at 600 nm (OD_600_ nm) reached 0.8. Protein expression was induced by adding 1 mmol L^−1^ isopropyl-β-thiogalactopyranoside for 4 h at 37 °C. Cell pellets were resuspended in buffer containing 20 mmol L^−1^ Tris-HCl (pH 7.5), 100 mmol L^−1^ NaCl, and 0.1% Tween-20, sonicated twice for 5 min at 4 °C and centrifuged at 20,000 × *g* for 30 min. The supernatant was incubated with glutathione Sepharose 4B (GE Healthcare) for 1 h at 4°C. The resin was washed five times with the same buffer, the glutathione *S*-transferase tag was cleaved by adding Precision Protease (GE Healthcare) and the sample was further incubated for 16 h at 4 °C. Cleaved proteins were recovered from the supernatants and stored at −80 °C.

### Affinity purification using the affinity nanobeads

Affinity nanobeads conjugating sesamin derivatives were prepared as previously described.^28,53^ Briefly, carboxylated sesamin derivatives SC1m, SC1 or curcumin were incubated at 1 mmol L^−1^ with equal amounts of *N*-hydroxysuccinimide and 1-ethyl-3-(3-dimethylaminopropyl) carbodiimide (Dojindo, Kumamoto, Japan) for 2 h at room temperature before reacting overnight with amino-modified affinity beads. For purification of binding proteins with sesamin derivatives, 0.2 mg of beads were equilibrated with binding buffer (20 mmol L^−1^ HEPES-NaOH (pH 7.9), 100 mmol L^−1^ NaCl, 10% glycerol, 0.1% NP40) and incubated with 1 mg mL^−1^ U937 cell extract at 4 °C for 1 h. Bound proteins were eluted with sodium dodecyl sulfate (SDS) loading buffer, separated using SDS-polyacrylamide gel electrophoresis (SDS-PAGE) and visualised using silver staining (Wako). Bound proteins were subjected to in-gel digestion using trypsin, and the peptide fragments were analysed using ESI-MS (Hitachi, NanoFrontier, Tokyo, Japan). For binding assays with recombinant ANX A1 proteins, full-length ANX A1 or deletion mutants (1 μg) were incubated with beads with or without 2 mmol L^−1^ EDTA (for apo-ANX A1) or 3 mmol L^−1^ CaCl_2_ (for Ca^2+^-bound ANX A1).

### ITC analyses

ITC experiments were performed at 298 K with ITC buffer (20 mmol L^−1^ Tris-HCl (pH 7.5), 100 mmol L^−1^ NaCl, 2% dimethyl sulfoxide) using a MicroCal iTC200 (Malvern). SC1 or SC2 were dissolved at 2 mmol L^−1^ with ITC buffer and titrated into 1 μmol L^−1^ of ANX A1 protein with or without 2 mmol L^−1^ EDTA (for apo-ANX A1) or 3 mmol L^−1^ CaCl_2_ (for Ca^2+^-bound ANX A1). The titration was performed by injecting 2 µL of the syringe solution at intervals of 120 s. The binding isotherms were analysed using SEDPHAT.^54^

### Docking simulation of ANX A1 binding with SC1

Docking simulation between ANX A1 and SC1 was performed by Kyoto Constella Technologies (Kyoto, Japan). Identification of the SC1-binding pocket of ANX A1 (PDB: 1MCX) was done using the software GROMACS^55^ and Fpocket.^56^ Docking was performed using AutoDock-Vina at 100 runs.^57^ The docking score was calculated as −8.830 kcal mol^−1^.

### Cell cultures

U937 cells were maintained in RPMI-1640 medium (Gibco/Thermo Fisher Scientific, MA, USA) supplemented with 10% fetal bovine serum. To generate a stable ANX A1-KD cell line, lentivirus vectors encoding a control or shRNA sequence targeting ANX A1 (Supplementary Methods) were transfected into 293FT cells. The lentivirus was prepared according to the manufacturer’s instructions (Invitrogen, MA, USA). U937 cells were infected with the lentivirus, and a stable cell line was selected by maintaining the cells in medium containing 10 μg mL^−1^ blasticidin (Invitrogen) for 1 week.

For enzyme-linked immunosorbent assay (ELISA) assays, U937 cells (control or ANX A1 KD) were seeded on 96-well plates and stimulated by adding 100 nmol L^−1^ phorbol myristate acetate (Sigma-Aldrich, MO, USA) for 24 h. The cells were treated for 12 h with LPS (100 ng mL^−1^) with or without adding the indicated sesamin, SC1, or SC2 and/or PD98059 concentrations. The cell culture supernatants were collected, and TNFα or ANX A1 production was measured using ELISA (TNFα: R&D Systems; ANX A1: USCN, Wuhan, China) according to the manufacturer’s instructions.

For analyses of ANX A1 phosphorylation, the U937 cells treated as above were lysed with NP40 lysis buffer (20 mmol L^−1^ Tris-HCl (pH 7.5), 150 mmol L^−1^ NaCl, 1% NP40), and the lysates (10 μg) were subjected to SDS-PAGE, followed by western blotting using antibodies against ANX A1 or phosphorylated (Ser27) ANX A1 (dilution: 1:1000).

### Generation of ANX A1-KO mice

ANX A1-deficient mice (C57BL/6J) were generated by homologous recombination at Trans Genic Inc. (Fukuoka, Japan), as shown in Fig. S4. The vector targeting *ANX A1* for deleting the coding region (exons 3–6) containing selectable neomycin-resistance (Neo) and PGK promoter genes was used. The targeting vector contained the 3.0 kbp of the 5′ arm flanking sequence and 4.5 kbp of the 3′ arm flanking sequence. The *PGK-Neor* gene was deleted by crossing with the CAG-flp mouse.^58^

### Analyses of CCl_4_-induced liver damage in mice

All protocols for animal procedures were approved by the Ethics Committee of Animal Experiments in accordance with the Internal Regulations on Animal Experiments at Keio University and Suntory Holdings Ltd, which are based on the Law for the Humane Treatment and Management of Animals (Law No. 105, 1 October 1973, as amended on 30 May 2014). Male C57BL/6J wild-type (WT) mice were purchased from CLEA Japan Inc. (Tokyo, Japan). WT and ANX A1-KO mice at 7 to 9 weeks of age received an intraperitoneal injection of vehicle (corn oil, 16.7 mL kg^−1^) or CCl_4_ (50 μL kg^−1^). To evaluate the effect of sesamin, vehicle (olive oil, 5 mL kg^−1^) or sesamin (25 mg kg^−1^) was orally administered twice at 1 h before and 7 h after CCl_4_ injection. To evaluate the effect of SC1, vehicle (ethanol dissolved in corn oil at a ratio of 1:19, 5 mL kg^−1^) or SC1 (50 mg kg^−1^) was intraperitoneally administered twice as mentioned above. Mice were euthanized after 12 or 24 h fasting using CCl_4_ injection before collecting blood and liver samples. Plasma AST, ALT, or LDH were measured using L-type AST J2, L-type ALT J2 or L-type LD J (Wako, Osaka, Japan), respectively. The liver tissues were fixed with 10% formalin, embedded in paraffin sectioned to 3–4 μm thickness and mounted on slides. Liver sections were de-paraffinized with xylene and re-hydrated with alcohols. The staining was performed with eosin solution for the cytoplasm and Mayer’s haematoxylin solution or monoclonal galectin-3 antibody (1:100, BioLegend, CA, USA). Slides were observed under a light microscope (BX52, Olympus, Japan), and images were analysed using LUMINA VISION (Mitani Corporation, Japan).

### Measurements of inflammatory proteins

Production of TNFα or MCP-1 in the plasma or liver was measured using a V-PLEX Proinflammatory Panel 1 Mouse Kit or U-PLEX Mouse MCP-1 assay Kit (Meso Scale Diagnostics, USA), respectively. Plasma or liver tissues homogenised in phosphate-buffered saline (Nacalai Tesque) were applied into the pre-coated plate with antibodies against target proteins before labelling with secondary antibodies conjugated to chemiluminescence. The signal intensity was measured using an analyser (SECTOR Imager 6000, Meso Scale Diagnostics, USA).

### Quantitative reverse transcription-PCR analyses

Total RNA of the liver was extracted using an RNeasy Mini Kit (Qiagen, USA), and the cDNAs were prepared using High-Capacity cDNA Reverse Transcription Kits (Thermo Fisher Scientific, USA). Quantitative reverse transcription-PCR was performed according to the manufacturer’s instructions using the TaqMan^®^ Gene Expression Assay System (Applied Biosystems, USA) with a 7900HT Fast Real Time PCR System (Applied Biosystems, USA). The levels of target mRNAs were normalised using the expression of the 18S ribosomal RNA. The primer IDs are listed in Supplementary Table 1.

### Statistics

Statistically significant differences were calculated using the unpaired Student’s *t* test. Data are presented as the mean ± SD or SE. *P* < 0.05 was considered statistically significant. Statistical analyses were performed using SPSS Statistics 25 software (IBM, USA).

### Reporting summary

Further information on research design is available in the Nature Research Reporting Summary linked to this article.

## Supplementary information

supplementary file

Reporting summary

## Data Availability

The authors declare that all data supporting the findings of this study are available in the paper and supplementary information.
