# Supplementary material for: Annexin A1 accounts for an anti-inflammatory binding target of sesamin metabolites
Source: NPJ Sci Food. 2020 Feb 20;4:4. doi: 10.1038/s41538-020-0064-6 (PMC7033200; doi:10.1038/s41538-020-0064-6)
Supplement: Supplementary file 1 — supplementary file [file 41538_2020_64_MOESM1_ESM.pdf]

Supplementary Information

Annexin A1 accounts for an anti-inflammatory binding target of sesamin metabolites

Yasuaki Kabe[1,2*†], Daisuke Takemoto[1,3*], Ayaka Kanai[1], Miwa Hirai[1], Yoshiko Ono[3], Sota Akazawa[3], Manabu Horikawa[4], Yoshinori Kitagawa[3], Hiroshi Handa[5], Tomohiro Rogi[3†], Hiroshi Shibata[3] and Makoto Suematsu[1†]

Supplementary Figures

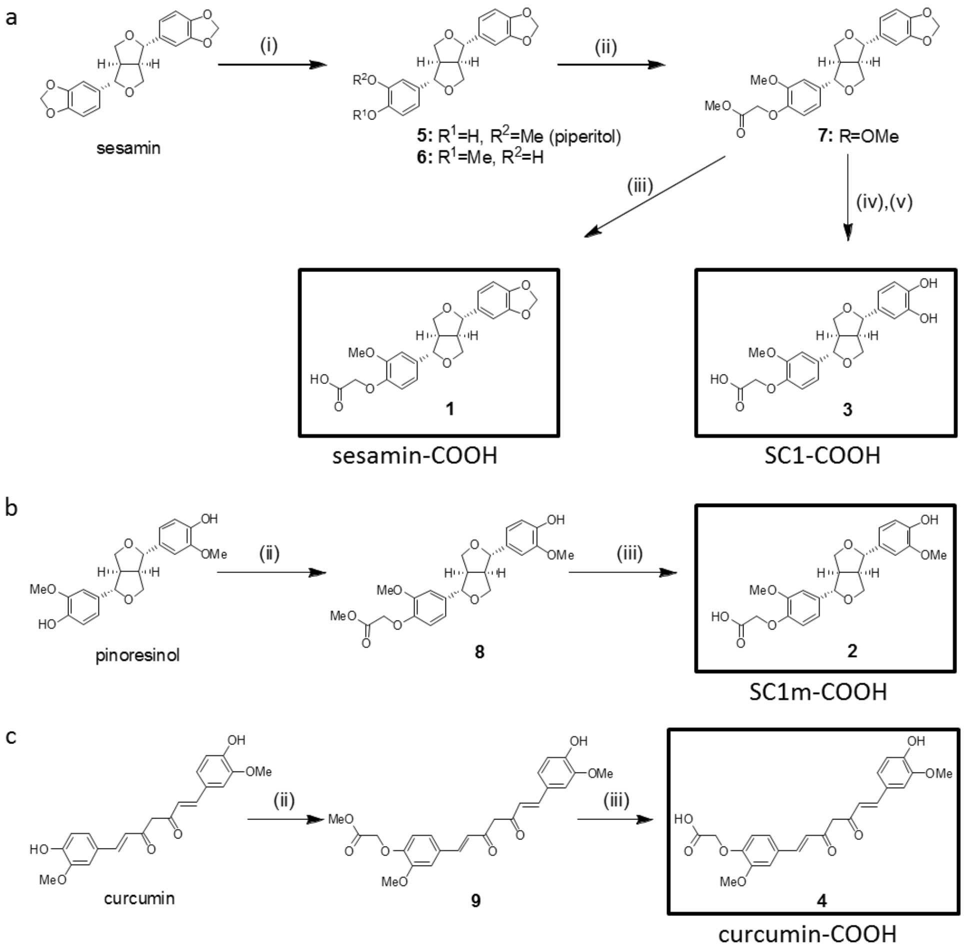

Supplementary Figure 1. Chemical synthesis of caroboxylated derivatives for conjugating affinity nanobeads

Scheme of chemical synthesis for carboxylated derivatives (**a**: sesamin-COOH, SC1-COOH, **b**: SC1m-COOH, **c**: curcumin-COOH). The synthetic conditions are indicated as (i) $^{i}Bu_2AlH$, toluene, reflux, 1 h, (ii) $BrCH_2CO_2Me$, K_2CO_3, DMF, rt, 3 h, (iii) 1N NaOHaq., THF, rt, 3 h, (iv) $Pb(OAc)_4$, benzene, 70°C, 1 h, (v) 1N LiOHaq., THF, rt, 1 h then 2 N $KHSO_4$ (pH 2), MeOH, rt, 15 min.

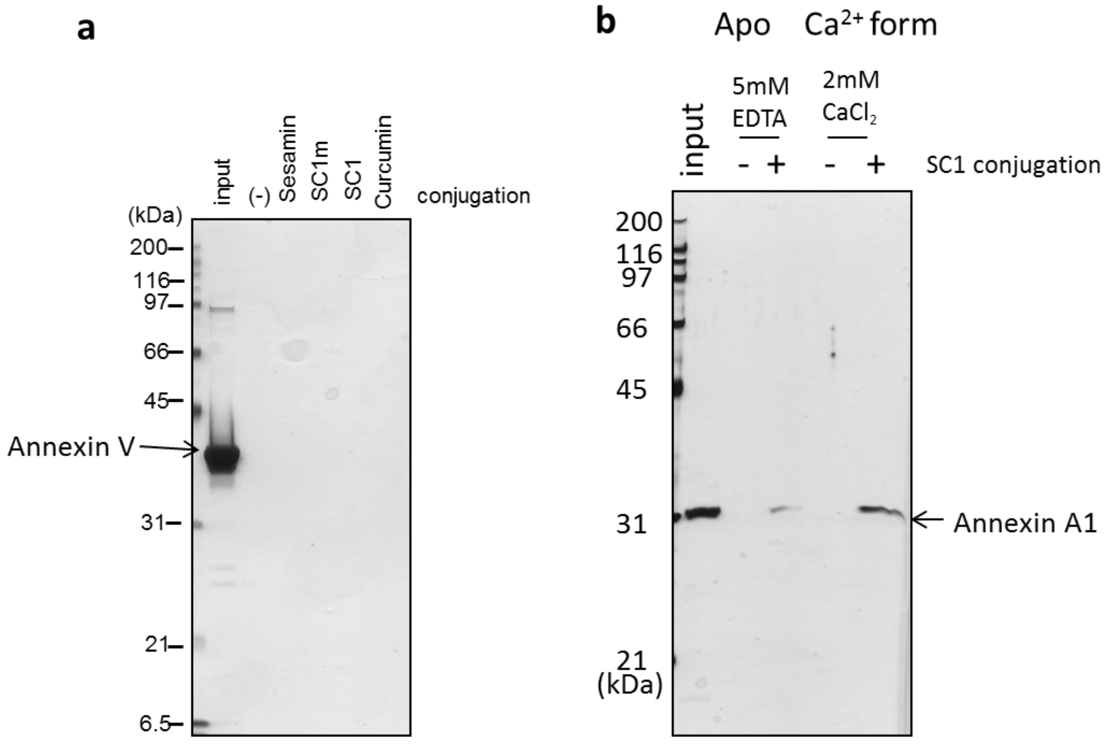

Supplementary Figure 2. Binding assay of recombinant proteins using affinity nanobeads

(**a**) Control, sesamin derivatives or curcumin-conjugated beads were incubated with recombinant annexin V protein (obtained from Abcam, USA), and bound protein analyzed with SDS-PAGE, followed by visualization via silver staining. (**b**) Control or SC1 conjugated beads were incubated with recombinant annexin A1 protein with buffer containing 2 mmol l^{-1} EDTA (Apo form) or 3 mmol l^{-1} $CaCl_2$ (Ca^{2+} form). Bound protein analyzed with SDS-PAGE, followed by visualization via silver staining.

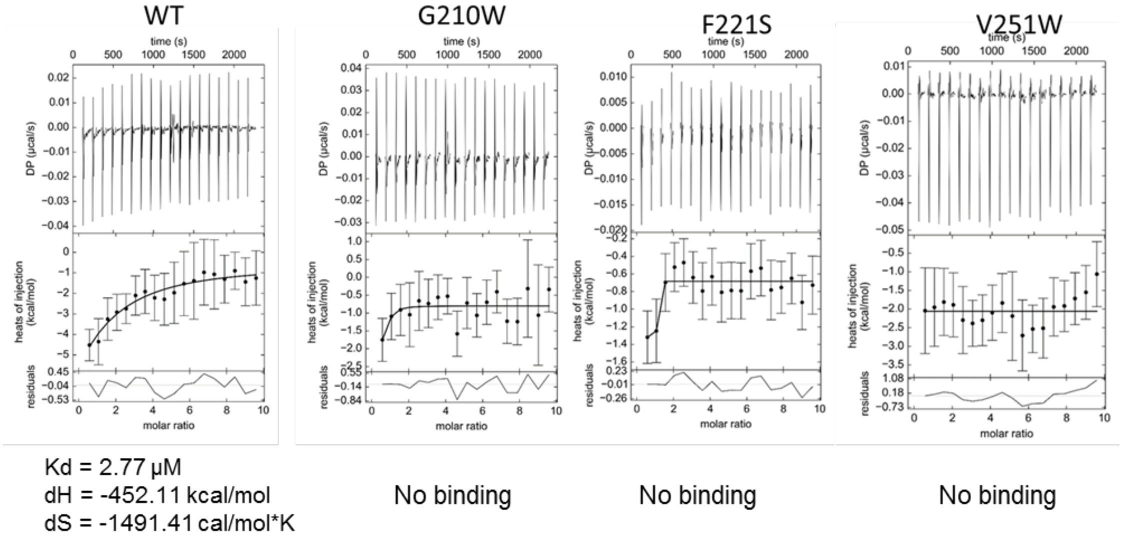

Supplementary Figure 3. Isothermal titration calorimetry analyses using ANX A1 mutant proteins

Analyses of binding affinity between SC1 and ANX A1 (wild type (WT) or point mutants (G210W, F221S, or V251W)) using isothermal titration calorimetry (ITC). The binding values (Kd, ΔH, and ΔS) were calculated using the SEDPHAT program.

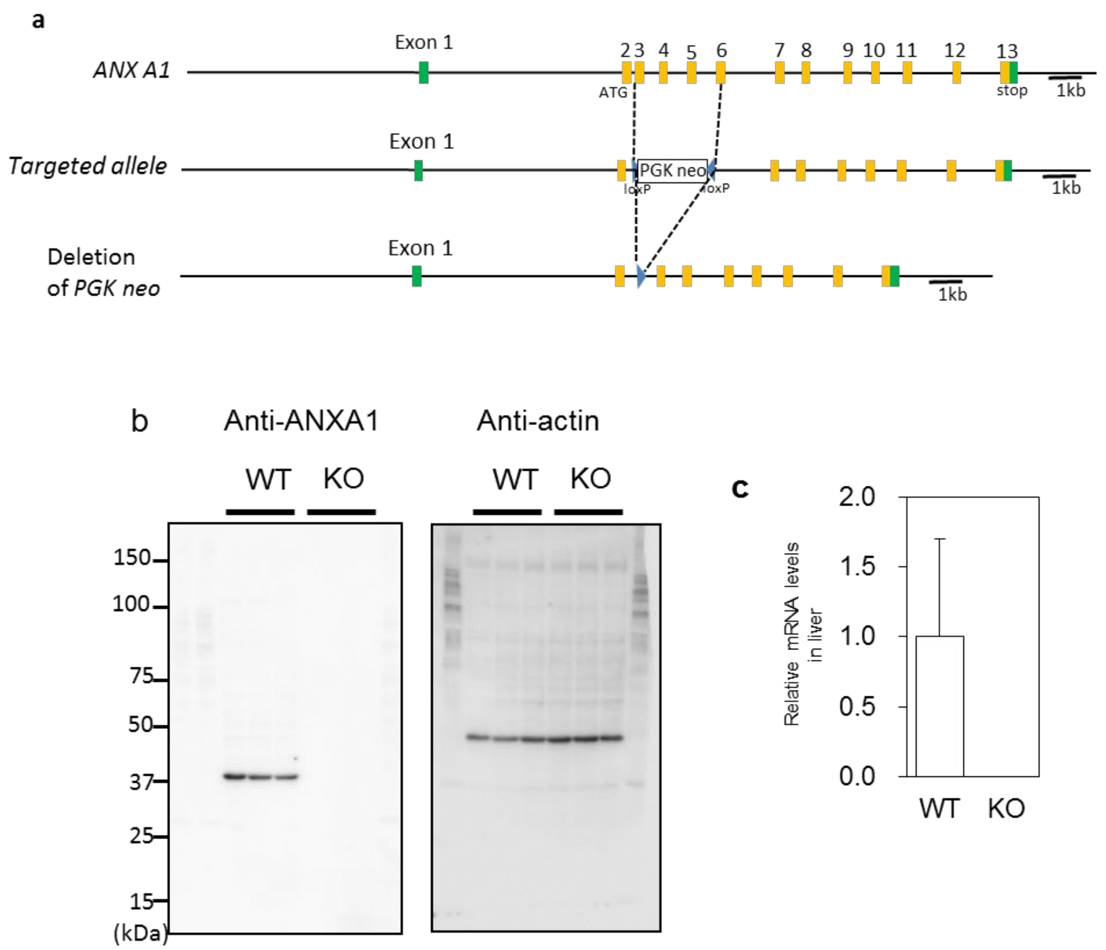

Supplementary Figure 4. Preparation of annexin A1 knockout mice

(**a**) Genomic organization of the wild-type and targeted alleles after homologous recombination at exons 3–6 of the mouse *ANX A1* gene. PGK; phosphoglycerate kinase I promoter, Neo; neomycin resistance gene. (**b, c**) Analyses of ANX A1 expression in the livers of ANX A1 KO mice. ANX A1 expression in the livers of WT or ANX A1 KO mice were measured by western blotting (**b**) or qPCR (**c**). Level of the mRNA expression is normalized by the expression of 18S rRNA. Data represent the mean ± SE. *n*=3 mice in each group.

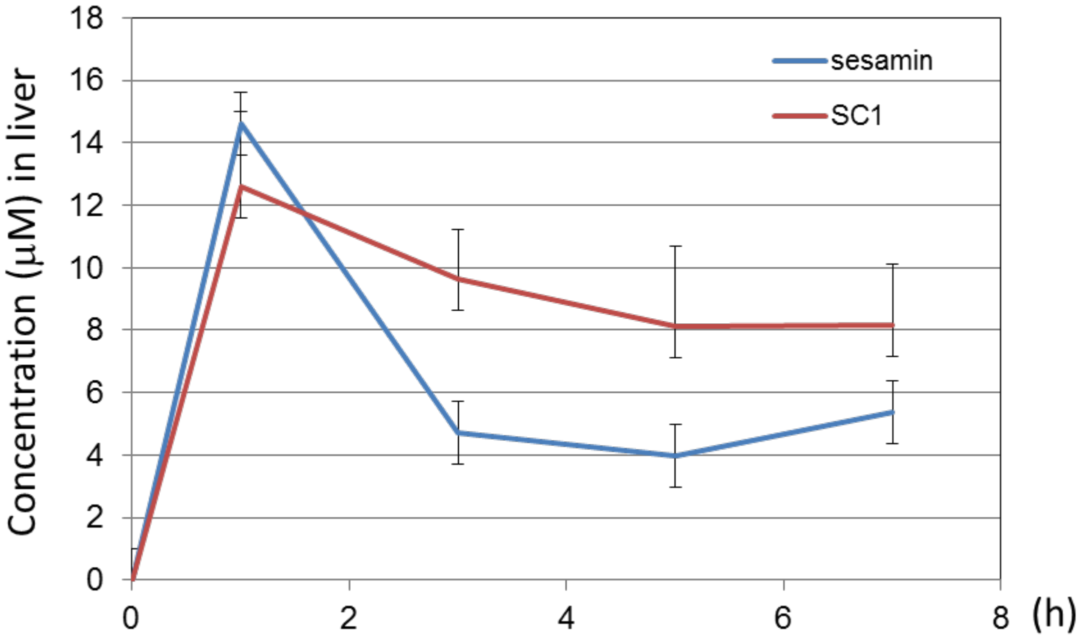

Supplementary Figure 5. Concentrations of sesamin and SC1 in the mouse liver

Sesamin (100 mg kg^{-1} body weight) was orally administered in C57BL/6J mice, and the liver was collected at the indicated time. Sesamin or SC1 in the liver was analyzed by LC-MS. Data represent the mean ± SE. *n*=3 mice in each group.

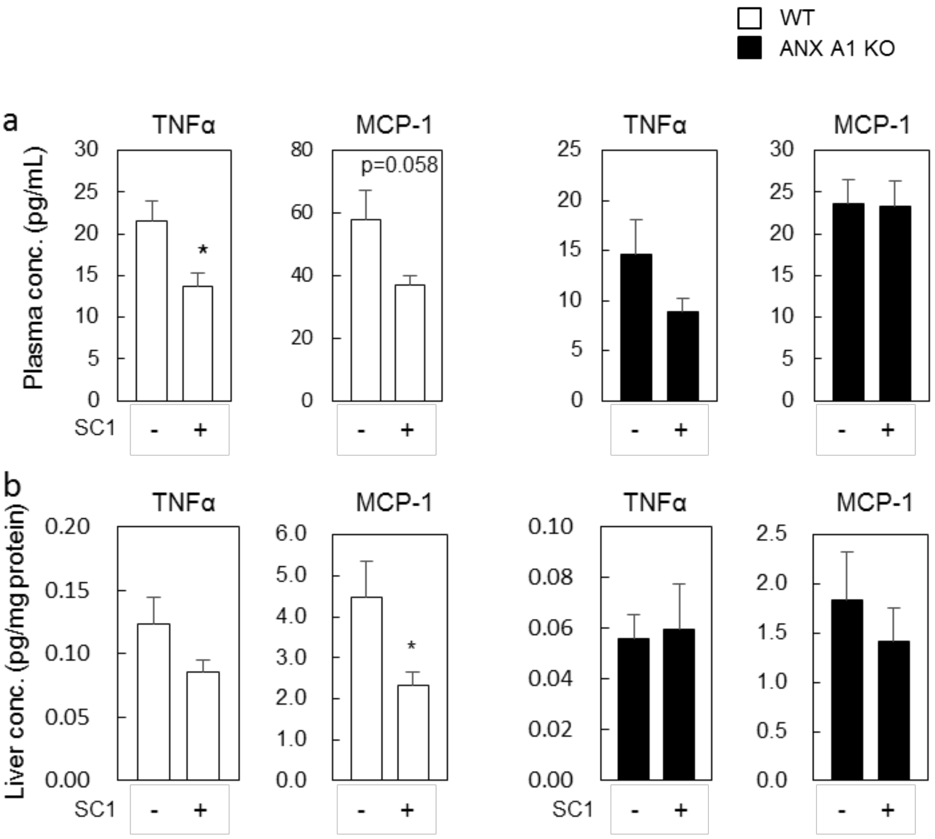

Supplementary Figure 6. Suppressive effect of SC1 against CCl4-induced inflammation

SC1 (50 mg kg^{-1} body weight) or vehicle was administrated intraperitoneally 2 times, at 1 h before and 7 h after CCl_4 administration. Twelve hours after CCl_4 administration, the plasma or liver was collected and production of TNFα or MCP1 protein in the plasma (**a**) or in the liver (**b**) was analyzed by ELISA. Data represent the mean ± SE. *n*=5-15 mice in each group. *p<0.05 using unpaired Student's t-test.

Fig. 3b

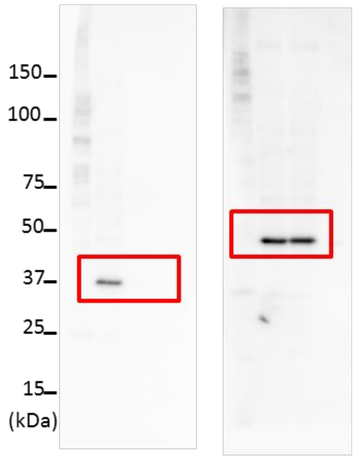

Fig. 4a

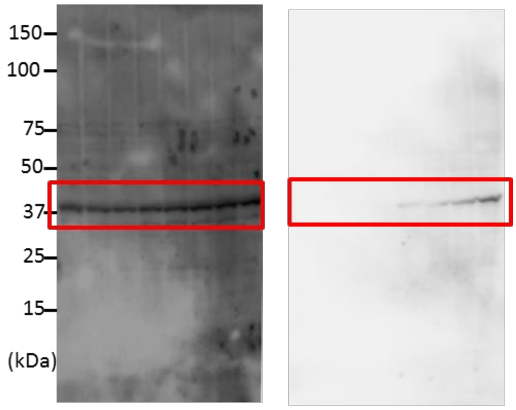

Fig. 4b

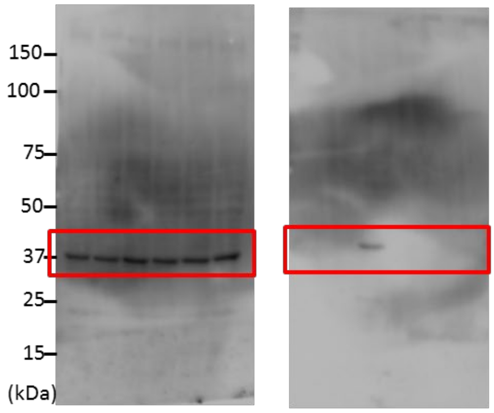

Supplementary Figure 7. Full images of western blotting

All blots derive from the same experiment and that they were processed in parallel.

Target protein	Gene name	Assay ID
18S (Endogenous Control)	Eukaryotic 18S rRNA	Hs99999901_s1
TNFα	tumor necrosis factor α	Mm00443258_m1
MCP-1	chemokine (C-C motif) ligand 2	Mm00441242_m1
Galectin3	lectin, galactose binding, soluble 3	Mm00802901_m1
Annexin A1	annexin A1	Mm00440218_m1
αSMA	actin, alpha 2, smooth muscle, aorta	Mm00725412_s1
Col1a1	collagen, type I, alpha 1	Mm00801666_g1

Supplementary Table 1. Primer IDs for qRT-PCR.

Supplementary Methods

Materials for chemical synthesis

Sesamin was purchased from ChromaDex, Inc. and purified by HPLC. Other reagents, including curcumin, were obtained from Nacalai tesque, TCI, or Sigma-Aldrich.

Chemical synthesis of carboxylated derivatives for conjugating affinity nanobeads

The synthesis of probe compounds (**1**, **2**, **3**, **4**) is shown in **Supplementary Fig S1**. Piperitol (**5**) synthesized from sesamin was converted through **7** into **1** by the general procedure to introduce an acetate moiety. Oxidation of **7** with lead (IV) acetate and the subsequent hydrolysis of **8** provided **3** as a probe molecule modelling the SC1 molecule. Based on the synthetic procedure of **1**, other probe molecules, **2** and **4**, were prepared through **9** and **10** from pinoresinol and curcumin, respectively.

Primers for plasmid constructions

ANX A1 full, forward:

5'-TTTGGATCTTATGGCAATGGTATCAGAATTCC-3'

ANX A1 full, reverse:

5'-TTTGTCGACTTAGTTTCCTCCACAAAGAGC-3'

ANX A1 44 a.a., forward:

5'-TTTGGATCCCCATCCTCGGATGTCGCTG-3'

ANX A1 201 a.a., reverse:

5'-TTTGTCGACATCAGCCAAGTCTTCATT-3'

ANX A1 184 a.a., forward:

5'-TTTGGATCCGCTAAGGGTGACCGATCT-3'

ANX A1 276 a.a., reverse:

5'-TTTGTCGACGAAAGCTGGTTTGCTTGT-3'

ANX A1 264 a.a., forward:

5'-TTTGGATCCGCTATCGTG AAGTGCGCCAC-3'

ANX A1 G210W, sense:

5'-CTTGTAAGAAGCATGGGAAAGGAGAAAG-3'

ANX A1 G210W, antisense:

5'-CTTTCTCCTTTCCCATGCTTCATACAAG-3'

ANX A1 F221S, sense:

5'-CAGACGTAAACGTGAGCATTACCATCCTTAC-3'

ANX A1 G210W, antisense:

5'-GTAAGGATGGTATTGCTCACGTTTACGTCTG-3'

ANX A1 V251W, sense:

5'-GACATGAACAAATGGCTGGACCTGGAG-3'

ANX A1 V251W, antisense:

5'-CTCCAGGTCCAGCCATTTGTTCATGTC-3'

ANX A1 shRNA, sense:

5'-GGATGTCGCTCCCTTGCATAATTCAAGAGATTATGCAAGGGA
GCGACATCCTTTT-3'

ANX A1 shRNA, antisense:

5'-GATCAAAAGGATGTCGCTCCCTTGCATAATTCAAGAGATTATGCAAGGGA
GCGACATCCCA-3'

Measurements of sesamin and SC1 in the liver using liquid chromatography-tandem mass spectrometry

Sesamin (100 mg kg^{-1}) was orally administered in C57BL/6J mice, and the livers were collected at the indicated times. The amounts of sesamin and SC1 in the liver tissue

homogenates were quantified using liquid chromatography-tandem mass spectrometry (LC-MS/MS). Briefly, a triple-quadrupole mass spectrometer equipped with an electrospray ionization (ESI) ion source (LCMS-8060, Shimadzu Corporation) was used in the positive-ESI and multiple reaction monitoring (MRM) modes. The samples were resolved on the Develosil C30 column (2 mmI.D. x 250 mmL, Nomura Chemical), using a step gradient with mobile phase A (5 mmol l^{-1} ammonium acetate, 0.1% formate) and mobile phase B (methanol with 5 mmol l^{-1} ammonium acetate, 0.1% formate) at ratios of 50:50 (0–5 min), 0:100 (5–40 min), and 50:50 (40–60 min), at a flow rate of 0.2 ml min^{-1}. MRM conditions of m/z 372>233 and m/z 359>233, were used for sesamin and SC1 detection, respectively.
